# Where Was the Editor?

**Published:** 1916-07-01

**Authors:** 


### THE EDITOR'S LETTER-BOX.

### Where was the Editor?

*To the Editor of* THE HOSPITAL.

SIR,—May I sound a note of surprise on reading the paragraph headed "A Lack of Patriotism at the Belgrave Hospital" in your issue of June 10? The law has set up Tribunals to hear and decide upon cases where employers feel that the work of some of their servants is of sufficient national importance to justify exemption from military service. There is nothing "to be thoroughly ashamed of" in making use of legal machinery which is provided for the purpose of hearing such applications. A hospital secretary may be as indispensable as thousands of other people to whom exemption has been given without challenge from the military authorities. Where a secretary is single-handed, as he often is in a small hospital like the Belgrave, it is exceedingly difficult to dispense with him; where he has an understudy, as in a larger hospital, it would not be so difficult.

As Editor of THE HOSPITAL you must know that the only trained secretaries over military age are all fully occupied, and that to meet the emergency men in retirement have cheerfully taken up the work again. History tells us that you are an ex-secretary; as such you know that an untrained woman cannot efficiently carry out a hospital secretary's duties. Is it possible that the Editor was having a holiday on June 10, and that, owing to the exigencies of the times, his substitute was the untrained person that the paragraph in question leads us to believe is considered good enough to fill an important office?—Yours faithfully,

HOSPITAL SECRETARY (aged forty-six).

*** After fourteen months' continuous work without a day's rebate the Editor was away resting by doctor's orders. His substitute was not "the untrained person," but a man of experience and knowledge who has done his part at the Front. The right of employers to appeal to the Tribunals is vested in them by statute, but the nearly universal practice is for employers to help the members of their staff to help the nation by providing material aid to enable every competent member to join the Colours free from anxiety for their dependants during war service.—ED. THE HOSPITAL.

### Advertisements in Voluntary Hospitals.

*To the Editor of* THE HOSPITAL.

